# Exploring the possible causal effects of cardiac blood biomarkers in dementia and cognitive performance: a Mendelian randomization study

**DOI:** 10.1007/s11357-023-00814-5

**Published:** 2023-05-13

**Authors:** Michelle H. Zonneveld, Stella Trompet, J. Wouter Jukema, Raymond Noordam

**Affiliations:** 1 Department of Internal Medicine, Section of Gerontology and Geriatrics, Leiden University Medical Center, Leiden, the Netherlands; 2 Department of Cardiology, Leiden University Medical Center, PO Box 9600, Leiden, 2300 RC the Netherlands; 3 Netherlands Heart Institute, Utrecht, the Netherlands

**Keywords:** Dementia, Cardiovascular disease, Cardiac biomarkers, Cognitive function, Older adults, Mendelian randomization

## Abstract

Prospective cohort studies have implied associations between blood levels of troponin T, troponin I, NT-proBNP, GDF15, dementia, and cognitive function, without providing evidence favoring possible causality. We aimed to assess the causal associations of these cardiac blood biomarkers with dementia and cognition using two-sample Mendelian randomization (MR). Independent genetic instruments (p < 5e^−7^) for troponin T and I, N-terminal pro B-type natriuretic peptide (NT-proBNP) and growth-differentiation factor 15 (GDF15) were obtained from previously-performed genome-wide association studies of predominantly European ancestry. Summary statistics for gene-outcome associations in European-ancestry participants, for the two-sample MR analyses, were obtained for general cognitive performance (*n* = 257,842) and dementia (*n* = 111,326 clinically diagnosed and “proxy” AD cases, and 677,663 controls). Two-sample MR analyses were performed using inverse variance-weighted (IWV) analyses. Sensitivity analyses to evaluate horizontal pleiotropy included weighted median estimator, MR-Egger, and MR using cis-SNPs only. Using IVW, we did not find evidence for possible causal associations between genetically influenced cardiac biomarkers with cognition and dementia. For example, per standard deviation (SD) higher cardiac blood biomarker, the odds ratio for risk of dementia was 1.06 (95%CI 0.90; 1.21) for troponin T, 0.98 (95%CI 0.72; 1.23) for troponin I, 0.97 (95%CI 0.90; 1.06) for NT-proBNP and 1.07 (95%CI 0.93; 1.21) for GDF15. Sensitivity analyses showed higher GDF15 was significantly associated with higher dementia risk and worse cognitive function. We did not find strong evidence that cardiac biomarkers causally influence dementia risk. Future research should aim at elucidating the biological pathways through which cardiac blood biomarkers associate with dementia.

**Supplementary Information:**

The online version contains supplementary material available at 10.1007/s11357-023-00814-5.

## Introduction

The role of cardiovascular disease (CVD) in the pathophysiology of dementia and Alzheimer’s Disease (AD) has been of growing interest in recent years [1]. Increasing epidemiological evidence implies a connection between CVD, cognitive decline and dementia; for example, diabetes, elevated total cholesterol and triglycerides in midlife have been associated with greater 20-year cognitive decline [2, 3]. In addition, various blood markers of cardiac (dys)function such as troponin T and I, N-terminal pro B-type natriuretic peptide (NT-proBNP) and growth-differentiation factor 15 (GDF15) have been linked to worse cognitive function and subsequent dementia [4–8]. A recent systematic review showed higher cardiac troponin T and I, markers of myocardial damage, have been found in individuals with worse memory, processing speed, executive function, as well as incident dementia and increased risk of dementia hospitalization [9]. Three prospective population-cohort studies report higher NT-proBNP, a marker of ventricular distension and in clinical practice used to diagnose congestive heart failure, to be significantly associated with an increased risk of dementia [10–12]. Elevated GDF15 concentrations in blood, as a marker of vascular stress and impaired endothelial function, have been linked to lower hippocampal volumes, greater white matter hyperintensity volume and poorer cognitive performance [6].

Though evidence regarding the association between these cardiac biomarkers and cognitive function is present, observational studies are not appropriate for causal inferences [13]. Mendelian randomization (MR) is an epidemiological approach used to evaluate potential causality between exposure and outcome [14], overcoming most limitations of multivariable-adjusted observational research such as confounding and reverse causation. The design employs genetic variations as instrumental variables, which are associated with the exposure, but not with confounders such as lifestyle, dietary and behavior factors [15]. Of particular interest, there is a growing need for valid, reliable risk factors for vascular cognitive impairment which could be employed to further our understanding of underlying pathology and potentially point to future therapeutic targets [6]. Previous MR studies report lower plasma levels of apolipoprotein (apoE) to contribute to higher risk of dementia [16], as well as genetically elevated blood pressure [17] and higher total cholesterol levels [18]. However, whether association between troponin-T, troponin-I, NT-proBNP and GDF15 and dementia is potentially causal remains unclear. Therefore, in this study, we aimed to evaluate the association of troponin-T, troponin-I, NT-proBNP and GDF15 with cognitive performance and risk of dementia using Mendelian Randomization.

## Methods

The data on which the results were based are summary-level data from previously performed genome-wide association studies. The reporting of this Mendelian randomization study was guided by the STROBE MR-checklist [19].

### Study design and data sources

We conducted a 2-sample MR analysis to test the association between genetic instrumental variables as proxy for the exposures (notably blood serum levels of troponin-T, troponin-I, NT-proBNP and GDF15) and outcome (cognitive performance, dementia, AD). There are 3 basic principles of MR: (1) genetic variants are associated with the exposure; (2) genetic variants are associated with the outcome exclusively through the exposure; and (3) genetic variants should be independent of any confounders. Data used in the present study are publicly available, and ethical approval and informed consent were obtained in each original study.

### Selection of genetic instrument variables

To study the associations between genetically influenced levels of cardiac blood biomarkers, cognitive performance and dementia, previously-published genome-wide association studies (GWAS) were used to identify the independent lead SNPs as genetic instrumental variables for the exposure. Only the largest of GWAS studies with predominantly European ancestry participants were used (Supplementary table 1) [20–22]. Yang et al. identified independent troponin T and I SNPs in a multiethnic population of 24,617 participants (18,590 from European ancestry, 3806 from African, 775 from Asian and 1446 from Hispanic ancestries) with mean age ranging from 47.13 to 76.21 years and proportions of women ranging from 50.8 to 65.1% [20]. The GWAS were adjusted for age, sex, study site (when applicable), and population-specific genotypic principal components. Salo et al. identified independent NT-proBNP SNPs in a population of 4,932 European-ancestry participants with mean age 46.18 years and 52.6% females, and included the same covariates as Yang et al. in addition to BMI. Last, GDF15 SNPs were identified in 21,758 European-ancestry participants, with mean age ranging from 18 to 86 years and proportions of females ranging from 19 to 66% [22], and were adjusted for age, sex, site, storage time, smoking, OLINK-plate number and population-specific genotypic principal components. The genetic variants were associated with the corresponding exposure below a *p*-value threshold of 5 × 10^–8^ or 5 × 10^–7^ in the case of troponin T and troponin I (all genetic instruments used in the present study are presented in Supplementary table 2). Resulting residuals were standardized before performing the GWAS.

### Data source for instrument-outcome associations

Summary statistics on the associations of the exposure-related SNPs with cognitive performance was extracted from the Cognitive Genomics Consortium (COGENT) [23], and exposure-related SNPs with dementia and Alzheimer’s Disease was extracted from the European Alzheimer & Dementia Biobank (EADB) consortium [24] (Table 1).

**Table 1:** Summary characteristics of COGENT and UK Biobank, and European Alzheimer and Dementia Biobank (EADB)

	N		Age (range), y		Women, range, %	
COGENT + UK Biobank	Cases	111,326	Cases	59.3 − 83.7	Cases	51.1 − 72.9
	Controls	677,663	Controls	44.4 − 75.7	Controls	52.0 − 71.0
EADB		257,841		16.0 − 102.0		52.0 − 71.0

*EADB* European Alzheimer & Dementia Biobank; *COGENT *Cognitive Genomics Consortium

The COGENT consortium performed a GWAS for cognitive performance in 257,841 individuals. These results were meta-analyzed with published results from UK Biobank [23]. Participants were of European descent and aged between 16 and 102 years. In COGENT, within each study included in the eventual meta-analysis, cognitive performance was measured as the score on the first unrotated component of the performance of at least 3 different neuropsychological tests. Population-specific genotypic principal components were included as covariates. In the UK Biobank, the verbal numerical reasoning test was used to measure fluid intelligence. The score was determined by number of questions answered correctly within 2 min (with a maximum of 13 questions). Estimates are reported in additive (per SNP) SD units.

The EADB consortium consists of 111,326 clinically diagnosed and “proxy” AD cases and 677,663 controls from 15 European countries. Cases were aged between 59.3 to 83.7 years, and controls were aged 50.7 to 82.8 years. Proxy cases were based on questionnaire data in which individuals were asked whether their parents had dementia. The two-phased GWAS meta-analysis included samples from ADGC, FinnGen and CHARGE consortia, and used population-specific genotypic principal components as covariates [24].

### Statistical analysis

All analyses were performed using R (version 4.2.1) statistical software (The R Foundation for Statistical Computing). MR analyses were performed using the R-based packages “TwoSampleMR,” “MRInstruments,” and “ieugwasr” (see https://mrcieu.github.io/TwoSampleMR/). Exposure and outcome SNPs were harmonized to remove palindromic SNPs and ensure effect estimates are aligned in the same direction. For the selection of genetic instruments for NT-proBNP, troponin T and I, data was used that already presented independent SNPs. Exposure SNPs associated with GDF15 were clumped before harmonization in order to obtain independent lead SNPs (minimal r2 = 0.001, window = 10,000 kb). Moreover, we performed separate analyses for cis-SNPs.

We used inverse variance-weighted (IVW) regression analysis for our primary analysis. This method assumes that there are no invalid genetic instruments causing horizontal/directional pleiotropy present in the SNPs used in the MR analysis. To overcome the issue of potentially using invalid instruments, MR-Egger regression analysis and weighted-median estimator were conducted to assess whether the results in the IVW regression were biased as a result of directional pleiotropy. The latter was performed only for cardiac blood biomarkers with > 3 genetic instruments. It must be noted that in the case of NT-proBNP, we were able to calculate the MR-Egger with dementia as outcome but not with cognitive function, as 1 SNP was missing from the outcome dataset. Furthermore, we used Cochran’s *Q* test statistic to examine the between-SNP heterogeneity and performed leave-one-out analysis (for cardiac blood biomarkers with > 2 genetic instruments) to observe whether one particular variant drives the association. F-statistics were calculated per SNP by squaring the beta divided by the standard error.

For each cardiac blood biomarker, we performed a power calculation (https://shiny.cnsgenomics.com/mRnd/). With power = 0.80, minimal effect sizes (beta) of cognitive performance ranged from 0.019 for NT-proBNP to 0.043 for troponin I, and minimal effect sizes (odds ratio) of dementia ranged from 1.031 for NT-proBNP to 1.230 for troponin I. Results for cognitive performance are presented as a beta (in standard deviations (SD)), with accompanying 95% confidence interval. For example, per SD higher cardiac blood biomarker, the change in cognitive performance is expressed in standard deviations. Results for dementia are expressed as odds ratio (OR) (standard error) per SD change in cardiac blood biomarker. Forest plots were designed using GraphPad Prism version 9.0.1 for Windows, GraphPad Software, San Diego, California, USA, www.graphpad.com.

### Sensitivity analyses

We repeated our analyses using cis-MR, which only uses genetic instruments that were located in the gene encoding for the protein in order to minimize possible pleiotropy or confounding. In addition, we have repeated our analyses on GDF15 and NT-proBNP using instruments that were derived from 54,306 participants from the UK Biobank (mean age 59.0 years and 51.2% women).

## Results

GWAS-summary statistics on dementia and cognitive function are presented in Table 1 (summary statistics on the cardiac blood biomarkers are presented in Supplementary table 1). Variance explained (*R*^2^) by instruments for each cardiac blood biomarker was calculated based on the derived summary statistics. This ranged from 0.17% for troponin I, to 3.31% for troponin T, 3.78% for GDF15 and to 8.74% for NT-proBNP. All SNPs had F-statistic of at least 10, which was considered sufficient for performing an MR-analysis (Supplementary table 2) [25].

### Cognitive performance

**Fig. 1:**
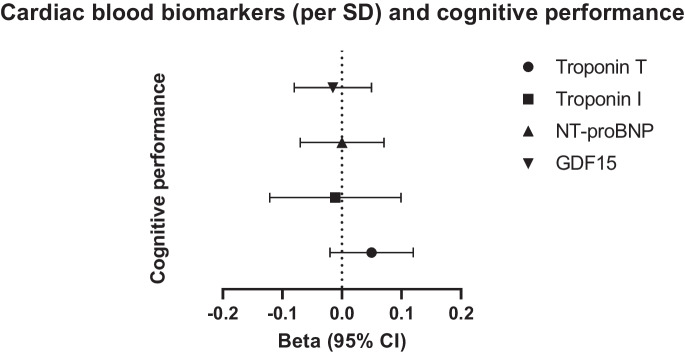
Results of MR analyses (inverse weighted variance estimator) between higher cardiac blood biomarkers and cognitive performance

We did not find evidence supporting causal associations between cardiac blood biomarkers and cognitive performance (Table 2 and Fig. 1). For example, using IVW regression analysis, we found per standard deviation (SD) higher troponin T, cognitive performance was 0.05 SD (95% CI − 0.02; 0.12, *p*-value = 0.19) higher. Similarly, per SD higher troponin I, NT-proBNP and GDF15, cognitive performance changed by − 0.013 SD (95% CI − 0.12; 0.10, *p*-value = 0.81), 0.001 SD (95% CI − 0.07; 0.07, *p*-value = 0.98), and − 0.02 SD (95% CI − 0.08; 0.05, *p*-value = 0.63), respectively. MR-Egger intercepts were calculated for troponin T and troponin I (*p*-value = 0.84 and *p*-value = 0.59, respectively). All estimates in the sensitivity analyses showed similar directionality as the IVW analysis. Exposure-outcome heterogeneity of each instrument evaluated by Cochran’s Q test statistics was detected in the case of troponin I (*p*-value = 0.021), NT-proBNP (*p*-value = 0.005) and GDF15 (*p*-value = 0.004), but not troponin T (*p*-value = 0.059). Leave-one-out plots suggested that the observed associations were unlikely to be driven by one specific SNP (Supplementary Fig. 1).

**Table 2:** Results of MR-Egger and weighted-median estimator analyses with cognitive performance as outcome

Exposure	Method	No. of IVs	Beta (95% CI)	SE	*P*-value	MR-Egger intercept (*p* value)	Heterogeneity *Q* value (*p* value)
Troponin T	IVW	10	0.05 (-0.02; 0.12)	0.036	0.185		0.059
	Weighted-median	10	0.03 (-0.03; 0.09)	0.03	0.462		
	MR-Egger	10	0.02 (-0.22; 0.26)	0.123	0.863	0.840	0.036
Troponin I	IVW	3	-0.013 (-0.12; 0.10)	0.056	0.810		0.021
	Weighted-median	3	0.007 (-0.07; 0.08)	0.037	0.840		
	MR-Egger	3	-0.34 (-1.18; 0.51)	0.43	0.580	0.589	0.026
NT-proBNP	IVW	2	0.001 (-0.07; 0.07)	0.037	0.982		0.005
	Weighted-median	2					
	MR-Egger	2				NA	NA
GDF15	IVW	2	-0.015 (-0.08; 0.05)	0.031	0.628		0.004
	Weighted-median	2					
	MR-Egger	2				NA	NA

*IVW* inverse variance weighted, *IVs* instrumental variables, *CI* confidence interval, *SE* standard error, *NA* not appropriate. Units for cognitive performance: per SD

### Dementia

**Fig. 2:**
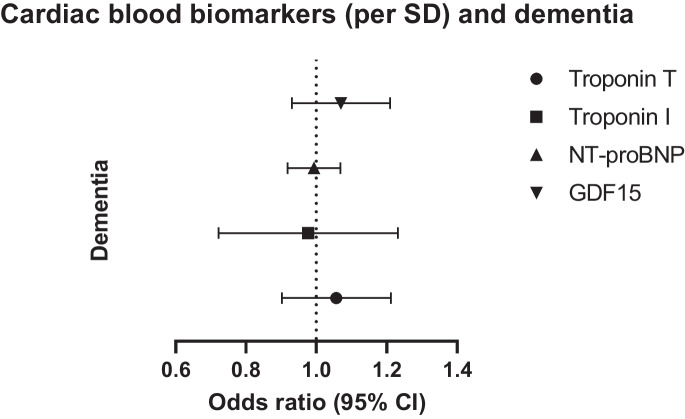
Results of MR analyses (inverse weighed variance estimator) between higher cardiac blood biomarkers and dementia

We observed no evidence for causal associations between cardiac blood biomarkers and dementia (Table 3 and Fig. 2). To illustrate; per SD higher troponin T, the odds ratio of dementia was 1.06 (95% CI 0.90; 1.21, *p*-value = 0.477). We found similar results for the other cardiac blood biomarkers: per SD higher troponin I, NT-proBNP and GDF15, the odds ratio of dementia was 0.98 (95% CI 0.72; 1.23, *p*-value = 0.86), 0.97 (95% CI 0.90; 1.06, *p*-value = 0.46), and 1.07 (95% CI 0.93; 1.12, *p*-value = 0.32), respectively. MR-egger intercepts did not deviate significantly from zero for troponin T (*p*-value = 0.481), troponin I (*p*-value = 0.811), and NT-proBNP (*p*-value = 0.640). All estimates in the sensitivity analyses showed similar directionality as the IVW analysis. Cochran’s Q statistic detected between-SNP heterogeneity in the case of GDF15 (*p*-value = 0.030), but not troponin T (*p*-value = 0.096), troponin I (*p*-value = 0.073), and NT-proBNP (*p*-value = 0.342). Leave-one-out plots suggested that the observed associations were unlikely to be driven by one specific SNP (Supplementary Fig. 2).

**Table 3:** Results of MR-Egger and weighted-median estimator analyses with dementia as outcome

Exposure	Method	No. of IVs	Odds ratio (95% CI)	Standard error	*P*-value	MR-Egger intercept (*p* value)	Heterogeneity Q value (*p* value)
Troponin T	IVW	10	1.06 (0.90; 1.21)	0.08	0.477		0.096
	Weighted-median	10	1.11 (0.95; 1.27)	0.08	0.210		
	MR-Egger	10	1.27 (1.11; 1.43)	0.26	0.386	0.481	0.085
Troponin I	IVW	3	0.98 (0.72; 1.23)	0.13	0.860		0.073
	Weighted-median	3	1.02 (0.79; 1.24)	0.12	0.900		
	MR-Egger	3	0.68 (0.45; 0.91)	1.185	0.802	0.811	0.029
NT-proBNP	IVW	2	0.97 (0.90; 1.06)	0.042	0.455		0.342
	Weighted-median	2					
	MR-Egger	2				NA	NA
GDF15	IVW	2	1.07 (0.93; 1.21)	0.07	0.320		0.03
	Weighted-median	2					
	MR-Egger	2				NA	NA

*IVW* inverse variance weighted, *IVs* instrumental variables, *CI* confidence interval, *NA* not appropriate

### Sensitivity analyses

Repeating the MR analyses using cis-SNPs only did not change results materially, except for GDF15. Here, higher GDF15 was now associated with worse cognitive function (beta 0.12, SE = 0.05, *p*-value = 0.013), but not with higher risk of dementia (OR 0.79, 95% CI 0.51; 1.07, *p*-value = 0.107).

We also repeated the analyses for GDF15 and NT-proBNP using SNP’s by Sun et al. (Supplementary table 3 and 4). The results for NT-proBNP remained unchanged. The results for GDF15 were similar to those of the cis-MR using data from the original dataset; per SD higher GDF15, cognitive performance was − 0.021 SD (95% CI − 0.04; − 0.001, *p*-value = 0.041) lower. Per SD higher GDF15, the odds ratio of dementia was 1.09 (95% CI 1.03; 1.15, *p*-value = 0.008).

## Discussion

In this two-sample MR study, we investigated the causal association between troponin T, troponin I, NT-proBNP, GDF15, and cognitive performance and dementia. Although the instrumental variables used in this study explain relatively low levels of genetic variance, our results show little evidence that genetically elevated cardiac blood biomarkers are unlikely to be causally associated with worse cognitive performance nor with increased risk of dementia.

Multiple population cohort studies have found associations between higher concentrations of cardiac biomarkers and worse cognitive outcomes. Both higher blood serum troponin T and I have been found to be associated with higher incidence of dementia [26], prevalence of cognitive impairment [27] and worse cognitive function [27–31]. In line, results from the prospective Rotterdam Study showed elevated blood serum NT-proBNP was associated with an increased risk of dementia, particularly for vascular dementia and Alzheimer’s dementia, independent of concomitant cardiovascular risk factors [12]. Higher cerebrospinal GDF15 levels were also associated with increased risk of incident dementia and lower total brain volumes, and was shown to associate with disease severity of Lewy Body dementia, independent of disease duration [6, 7]. However, inconsistent trends have also been reported by other cohort studies. For example, higher troponin was not associated with risk of Alzheimer’s Disease [26], and another study reports neither troponin T nor NT-proBNP were associated with accelerated cognitive decline over 15-years follow-up [32]. These inconsistencies may be explained by differences in sample size, study design (cross-sectional versus prospective) and use of cognitive function tests. Nevertheless, majority of evidence from epidemiological studies point towards an association between cardiac blood biomarkers, cognitive performance and dementia [9], although we were not able to provide any evidence favoring a possible causal relationship between troponin T, troponin I and NT-proBNP and cognitive function and dementia. We did show higher GDF15 to be associated with worse cognitive function and higher risk of dementia. However, the very modest effect sizes and still relatively low number of genetic instruments used nuances this finding and no proper statistical corrections for potential pleiotropy could be made. Another option to further test the validity of the used instrumental variables would be to perform a colocalization analysis, which is performed more frequently in combination with cis-MR, but would require full summary-level data which was not available at the time of the study.

Various biological pathways can potentially explain the evidence for observed associations between cardiac blood biomarkers and dementia risk in cohort studies. The markers used in the present study are all markers that mainly increase in serum concentration as a result of cardiac damage, whereas other cardiovascular risk factors are already present or elevated in the preclinical phase, such as higher blood pressure or increased lipoprotein(a) concentration.. For example, previous MR studies have demonstrated a causal association between higher blood pressure with worse cognitive function and higher risk of Alzheimer’s disease [33, 34]. Another MR study reported a causal relationship between lipoprotein(a) (Lp(a)) and dementia [35, 36]. Interestingly, increased concentrations of Lp(a), a well-established risk-factor of coronary heart disease [37], were found to cause a lower risk of AD [36]. This is in line with results from some prospective cohort studies, as Lp(a) level has shown to be protective of future dementia risk in a middle-aged Finnish male population [35] and with slower cognitive decline [38, 39]. However, other studies do not corroborate these findings [40, 41]. Lp(a) is a lipoprotein composed of a LDL-like core and glycoprotein Apo(a), and has pro-atherosclerotic and inflammatory effects, contributing to the onset ischemic vascular events [37]. Mechanisms by which elevated Lp(a) reduce risk of AD remain unclear. However, Lp(a) may function as a mediator in pathways related to elevated cardiac blood biomarker concentrations as well as changes in cognitive function. Perhaps it is not cardiovascular damage itself that is connected with an increased risk of dementia, but the preclinical phase. This may explain why the markers used in the present study, which are elevated following cardiovascular damage, do not show a strong association with worse cognitive function or higher risk of dementia, as opposed to markers which are elevated before cardiovascular damage.

Second, in line with the pro-inflammatory effects of Lp(a), biomarkers of immunity and inflammation may also participate in the pathway between cardiac biomarkers and dementia. Troponin T, troponin I, NT-proBNP, and GDF15 are markers of vascular damage and their release can trigger an inflammatory cascade. An observational study of over 5000 participants found higher levels of IL-6 and CRP to be associated with worse cognitive function and steeper cognitive decline [42, 43]. Moreover, a MR study using data from the FinnGen cohort demonstrated higher CRP to be associated with an increased risk of vascular dementia [44]. On the other hand, a recent MR study using the same outcome data as the present study found genetically predicted elevated levels of IL-8 were associated with better cognitive performance, although the results were modest [45]. Inflammaging, a phenomenon describing the intersection between low-grade chronic inflammation and ageing, contributes to the pathogenesis of age-related diseases such as dementia and cardiovascular disease [46]. Inflammatory molecules can freely pass the blood–brain barrier and cause cerebral tissue damage through neurodegeneration, as seen in mice overexpressing pro-inflammatory cytokines [47]. However, the directionality of the relationship between the immune system and dementia is ambiguous as it is difficult to distinguish cause-and-effect.

Third, the roles of other cardiac blood biomarkers, such as myoglobin and creatine kinase-MB (CK-MB, cardiac specific fraction), may also be of importance. Myoglobin and CK-MB are also sensitive markers of acute myocardial infarction (AMI). Myoglobin is found in both cardiac and skeletal muscle, and is involved in transport and binding of oxygen. Its concentration increases 2–3 h following an AMI, making it the first cardiac biomarker to increase (in comparison; troponin is measurable after 4 − 9 h) [48]. In line, CK-MB is an enzyme specific for cardiac tissue and is involved in energy expenditure. Its serum levels also increase 3–6 h following an AMI, similar to myoglobin. Although troponin is the golden standard for clinical diagnosis of AMI, myoglobin and CK-mb appear to embody comparable pathophysiological significance. Strikingly, there is no literature available for the association between myoglobin and CK-MB with cognitive function and dementia. In addition, genome-wide association studies have not (yet) been performed for neither myoglobin nor CK-MB. Future research is warranted into the contributions of these two markers in cognitive changes.

To the best of our knowledge, this is the first MR study between troponin T, troponin I, NT-proBNP, GDF15, cognitive performance and dementia. The two-sample design ameliorates the shortcomings of observational studies such as residual confounding and reverse causation. However, there are some limitations that must be considered when interpreting results of this study. First, although the sample size of our outcomes were large (> 250,000 cognitive performance and > 100,000 cases of dementia), the study populations could be seen as relatively young for worse cognitive performance and dementia occurrence (age range starting at 16 years). The study populations consist predominantly (although not entirely) of European ancestry participants, potentially leading to bias due to population stratification, limiting extrapolation to other (non-European) populations. In addition, blood serum measurements of the biomarkers may not be fully representative of concentrations in the brain and as a result cannot reflect the true effects. Troponin I also displayed low explained variance (0.17%) which can explain the relatively low statistical power. Furthermore, there were only few genetic instruments associated with each cardiac blood biomarker, each with a relatively modest variance explained (between 0.17% and 8%). This was also the case when we repeated the analyses using genetic instruments derived from a larger study sample from the UK Biobank. This may explain why our results may not meet all of the minimal effect sizes needed. The limited number of available genetic variants as instrumental variables also prohibited further sensitivity analyses. Instead, we performed a PheWAS for genetic variants of GDF15 to evaluate whether the included SNPs could possibly have pleiotropic effects (https://www.ebi.ac.uk/gwas/). The GDF15 variants used in the present study were associated with other phenotypes such as body mass index in addition to GDF15 concentrations; however, it is rather unlikely that this could be an explanatory pleiotropic pathway. However, in spite of the limitations, our findings did not change when replicating our analyses using data from a larger UK Biobank cohort (> 50,000 participants) nor when we performed cis-MR analyses using only genetic instruments located in the gene encoding for the protein, limiting the influence of confounding.

In summary, our study did not provide evidence fully supporting a causal association between troponin T, troponin I, NT-proBNP, and GDF15 with cognitive performance and dementia. Further research should aim to elucidate causal pathways by which cardiac biomarkers associate with cognitive functioning and dementia.

### Supplementary Information

Below is the link to the electronic supplementary material.

Supplementary file1 (DOCX 620 KB)

## Data Availability

Data (summary statistics) are available from the original sources (see heading "Data source for instrument-outcome associations").
