# Supplementary material for: Exploring the possible causal effects of cardiac blood biomarkers in dementia and cognitive performance: a Mendelian randomization study
Source: GeroScience. 2023 May 13;45(6):3165–74. doi: 10.1007/s11357-023-00814-5 (PMC10643774; doi:10.1007/s11357-023-00814-5)
Supplement: Supplementary file 1 — Supplementary file1 (DOCX 620 KB) [file 11357_2023_814_MOESM1_ESM.docx]

**Supplementary table 1.** Summary statistics of cardiac blood biomarkers genome-wide association studies.

|  | **Biomarker** | **N** | **Ethnicity (n)** | **Mean age (range) in years** | **Proportion of women (%)** |
| --- | --- | --- | --- | --- | --- |
| **Yang et al.** | Troponin I  Troponin T | 24,617 | 18,590 European  3,806 African  775 Asian  1446 Hispanic | 47.13-76.21 | 40.8-61.5 |
| **Salo et al.** | NT-proBNP | 4,932 | European | 46.18 | 52.6 |
| **Folkersen et al.** | GDF15 | 21,758 | European | 18-86 | 19-66 |
| **Sun et al.** | GDF15  NT-proBNP | 54,306 | 47,952 Caucasian  597 Asian  3801 Black  543 Chinese | 59.0 | 51.2 |

**Supplementary table 2.** List of genetic instruments of cardiac blood biomarkers.

| **SNP** | **Trait** | **Nearest gene** | **Chromosome** | **Location** | **Effect allele** | **Other allele** | **EAF** | **Exposure beta** | **SE** | **P-value** | **F-statistic** |
| --- | --- | --- | --- | --- | --- | --- | --- | --- | --- | --- | --- |
| rs9944895 | Troponin T | *BCL2* | 18 | 18:63192741 | c | g | 0.69 | 0.07 | 0.01 | 1.05E-15 | 49.00 |
| rs3737882 | Troponin T | *PPFIA4* | 1 | 1:203065827 | c | g | 0.82 | 0.06 | 0.01 | 2.8E-09 | 36.00 |
| rs28581409 | Troponin T | *TRAM1* | 8 | 8:70494824 | a | g | 0.34 | -0.05 | 0.01 | 6.63E-09 | 25.00 |
| rs75244633 | Troponin T | *SYNPO2* | 4 | 4:118958433 | t | c | 0.02 | 0.14 | 0.03 | 1.44E-07 | 21.78 |
| rs146737477 | Troponin T | *TTLL7* | 1 | 1:83297598 | a | g | 0.03 | -0.25 | 0.05 | 1.65E-07 | 25.00 |
| rs12506869 | Troponin T | *DDIT4L* | 4 | 4:100079830 | a | g | 0.26 | -0.05 | 0.01 | 2.13E-07 | 25.00 |
| rs199460 | Troponin T | *NSF* | 17 | 17:46687409 | a | c | 0.74 | -0.05 | 0.01 | 3.07E-07 | 25.00 |
| rs17618762 | Troponin T | *LINC00954;TTC32* | 2 | 2:19646343 | a | g | 0.93 | -0.09 | 0.02 | 3.37E-07 | 20.25 |
| rs13341435 | Troponin T | *APOH* | 17 | 17:66254487 | a | g | 0.06 | 0.08 | 0.02 | 5.61E-07 | 16.00 |
| rs1192168 | Troponin T | *SART1* | 11 | 11:65963474 | t | g | 0.50 | 0.04 | 0.01 | 7.27E-07 | 16.00 |
| rs9899998 | Troponin T | *NF1;RAB11FIP4* | 17 | 17:31383996 | a | g | 0.06 | -0.19 | 0.04 | 8.22E-07 | 22.56 |
| rs4922982 | Troponin T | *ANO5* | 11 | 11:22215819 | t | c | 0.31 | -0.04 | 0.01 | 9.21E-07 | 16.00 |
| rs116819086 | Troponin T | *RARB* | 3 | 3:25407513 | c | g | 0.04 | -0.24 | 0.05 | 9.23E-07 | 23.04 |
| rs7915720 | Troponin I | *VCL;AP3M1* | 10 | 10:74014381 | a | g | 0.32 | 0.07 | 0.012 | 5.51E-08 | 34.03 |
| rs2915700 | Troponin I | *VPS41;POU6F2* | 7 | 7:38944677 | a | g | 0.17 | 0.09 | 0.019 | 7.97E-07 | 22.44 |
| rs26742 | Troponin I | *MYO10* | 5 | 5:16664660 | a | g | 0.57 | -0.06 | 0.012 | 9.45E-07 | 25.00 |
| rs61761991 | NT-proBNP | *NPPB* | 1 | 1:11858387 | t | c | 0.029 | -0.766 | 0.044 | 1.72E-51 | 303.08 |
| rs12406089 | NT-proBNP | *NPPB* | 1 | 1:11861124 | g | c | 0.291 | 0.201 | 0.0172 | 8.31E-48 | 136.56 |
| rs1227734 | GDF15 | *LRRC25* | 19 | 19:18390224 | t | C | 0.136 | 0.369 | 0.013 | 9.92E-177 | 805.69 |
| rs4808135 | GDF15 | *GDF15* | 19 |  | c | g | 0.787 | 0.074 | 0.011 | 1.97E-11 | 45.26 |

Information is gathered from the original GWAS studies.

**Supplementary table 3.** Replication of analyses using exposure set by Sun et al in UK Biobank. Results of ***pan*** MR-Egger and weighted-median estimator analyses.

| **Cognitive performance** | | | | | | | | |
| --- | --- | --- | --- | --- | --- | --- | --- | --- |
| **Exposure** | | **Method** | **No. of IVs** | **Beta (95% CI)** | **SE** | **P-value** | **MR-Egger intercept (p value)** | **Heterogeneity *Q* value (p value)** |
|  | NT-proBNP | IVW | 4 | -0.001 (-0.03; 0.03) | 0.016 | 0.943 |  | 0.033 |
|  | | Weighted-median | 4 | -0.001 (-0.02; 0.02) | 0.010 | 0.959 |  |  |
|  | | MR-Egger | 4 | -0.001 (-0.08; 0.08) | 0.043 | 0.976 | 0.994 | 0.078 |
|  | GDF15 | Wald ratio | 1 | -0.021 (-0.04; -0.001) | 0.01 | 0.041 | NA | NA |
| **Dementia** | | | | | | | | |
| **Exposure** | | **Method** | **No. of IVs** | **Odds ratio (95% CI)** | **Standard error** | **P-value** | **MR-Egger intercept (p value)** | **Heterogeneity Q value (p value)** |
|  | NT-proBNP | IVW | 4 | 0.98 (0.88; 1.08) | 0.05 | 0.666 |  | 0.033 |
|  | | Weighted-median | 4 | 0.97 (0.91; 1.03) | 0.03 | 0.283 |  |  |
|  | | MR-Egger | 4 | 0.82 (0.68; 0.96) | 0.07 | 0.099 | 0.101 | 0.078 |
|  | GDF15 | IVW | 2 | 1.09 (1.03; 1.15) | 0.03 | 0.008 | NA | NA |
|  | | Weighted-median |  |  |  |  |  |  |
|  | | MR-Egger |  |  |  |  |  |  |

Abbreviations: IVW = inverse variance weighted, IVs = instrumental variables, CI = confidence interval, SE = standard error, NA = not appropriate. Units for cognitive performance: per SD.

**Supplementary table 4.** Replication of analyses using exposure set by Sun et al in UK Biobank. Results of ***cis*** MR-Egger and weighted-median estimator analyses.

| **Cognitive performance** | | | | | | | | |
| --- | --- | --- | --- | --- | --- | --- | --- | --- |
| **Exposure** | | **Method** | **No. of IVs** | **Beta (95% CI)** | **SE** | **P-value** | **MR-Egger intercept (p value)** | **Heterogeneity *Q* value (p value)** |
|  | NT-proBNP | Wald ratio | 1 | -0.003 (-0.02; 0.023) | 0.01 | 0.741 | NA | NA |
|  | GDF15 | Wald ratio | 1 | -0.021 (-0.04; -0.001) | 0.01 | 0.041 | NA | NA |
| **Dementia** | | | | | | | | |
| **Exposure** | | **Method** | **No. of IVs** | **Odds ratio (95% CI)** | **Standard error** | **P-value** | **MR-Egger intercept (p value)** | **Heterogeneity *Q* value (p value)** |
|  | NT-proBNP | Wald ratio | 1 | 0.95 (0.89; 1.01) | 0.03 | 0.083 | NA | NA |
|  | GDF15 | Wald ratio | 1 | 1.09 (1.03; 1.15) | 0.03 | 0.008 | NA | NA |

Abbreviations: IVW = inverse variance weighted, IVs = instrumental variables, CI = confidence interval, SE = standard error, NA = not appropriate. Units for cognitive performance: per SD.

**Supplementary Figure 1.** Leave-one-out plots for cognitive performance.


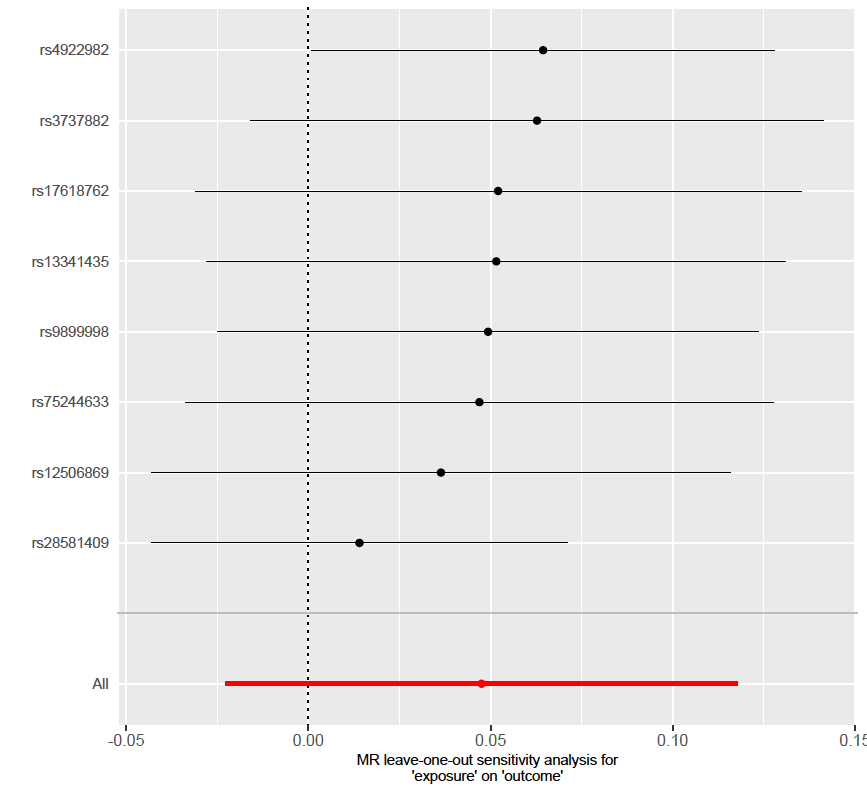

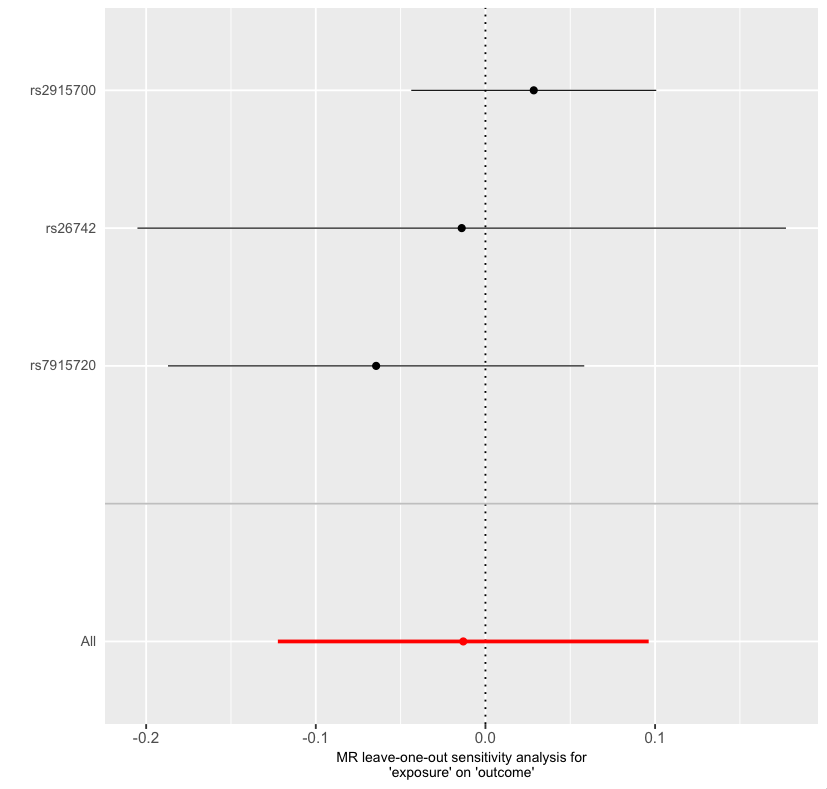


Left = troponin T SNPs, right = troponin I SNPs.

**Supplementary Figure 2.** Leave-one-out plots for dementia.


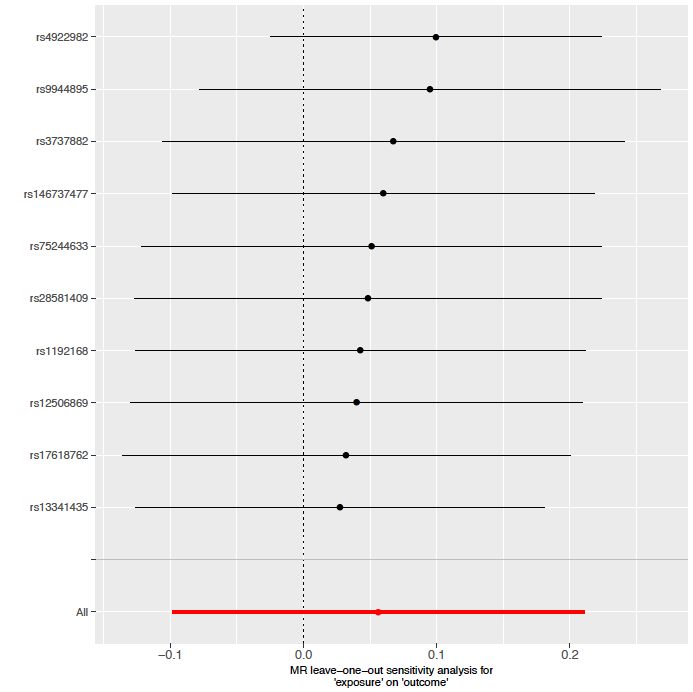

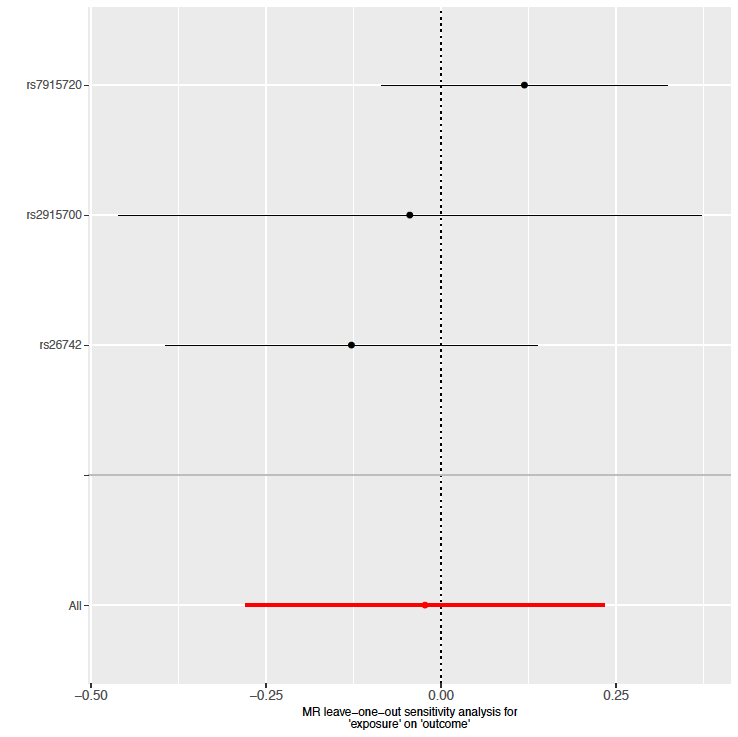

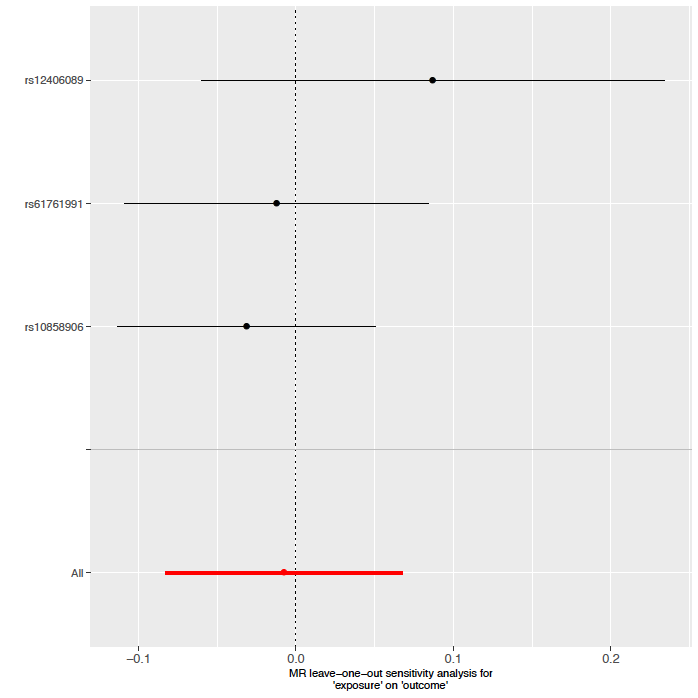


Left = troponin T, middle = troponin I, right = NT-proBNP.
